# Supplementary material for: Potential contribution of vaccination uptake to occupational differences in risk of SARS-CoV-2: analysis of the ONS COVID-19 Infection Survey
Source: Occup Environ Med. 2023 Dec 12;81(1):34–9. doi: 10.1136/oemed-2023-108931 (PMC10850636; doi:10.1136/oemed-2023-108931)
Supplement: Supplementary data [file oemed-2023-108931supp001.pdf]

Supplementary Tables

SOC2010	NEW OCCUPATIONAL GROUPINGS	Occ_subgroups
1115	Chief executives and senior officials	other workers-office based
1116	Elected officers and representatives	other workers-office based
1121	Production managers and directors in manufacturing	other workers-office based
1122	Production managers and directors in construction	other workers-office based
1123	Production managers and directors in mining and energy	other workers-office based
1131	Financial managers and directors	other workers-office based
1132	Marketing and sales directors	other workers-office based
1133	Purchasing managers and directors	other workers-office based
1134	Advertising and public relations directors	other workers-office based
1135	Human resource managers and directors	other workers-office based
1136	Information technology and telecommunications directors	other workers-office based
1139	Functional managers and directors n.e.c.	other workers-office based
1150	Financial institution managers and directors	other workers-office based
1161	Managers and directors in transport and distribution	other workers-office based
1162	Managers and directors in storage and warehousing	other workers-office based
1171	Officers in armed forces	police and protective services
1172	Senior police officers	police and protective services
1173	Senior officers in fire, ambulance, prison and related services	police and protective services
1181	Health services and public health managers and directors	healthcare-office based
1184	Social services managers and directors	social care
1190	Managers and directors in retail and wholesale	retail
1211	Managers and proprietors in agriculture and horticulture	other workers-non-office based
1213	Managers and proprietors in forestry, fishing and related services	other workers-non-office based
1221	Hotel and accommodation managers and proprietors	hospitality
1223	Restaurant and catering establishment managers and proprietors	hospitality
1224	Publicans and managers of licensed premises	hospitality
1225	Leisure and sports managers	other workers-office based
1226	Travel agency managers and proprietors	other workers-office based
1241	Health care practice managers	healthcare-office based
1242	Residential, day and domiciliary care managers and proprietors	social care
1251	Property, housing and estate managers	other workers-non-office based

1252	Garage managers and proprietors	other workers-non-office based
1253	Hairdressing and beauty salon managers and proprietors	personal care
1254	Shopkeepers and proprietors – wholesale and retail	retail
1255	Waste disposal and environmental services managers	sanitation services
1259	Managers and proprietors in other services n.e.c.	other workers-office based
2111	Chemical scientists	other workers-non-office based
2112	Biological scientists and biochemists	other workers-non-office based
2113	Physical scientists	other workers-non-office based
2114	Social and humanities scientists	other workers-non-office based
2119	Natural and social science professionals n.e.c.	other workers-office based
2121	Civil engineers	other workers-non-office based
2122	Mechanical engineers	other workers-non-office based
2123	Electrical engineers	other workers-non-office based
2124	Electronics engineers	other workers-non-office based
2126	Design and development engineers	other workers-non-office based
2127	Production and process engineers	other workers-non-office based
2129	Engineering professionals n.e.c.	other workers-non-office based
2133	IT specialist managers	other workers-office based
2134	IT project and programme managers	other workers-office based
2135	IT business analysts, architects and systems designers	other workers-office based
2136	Programmers and software development professionals	other workers-office based
2137	Web design and development professionals	other workers-office based
2139	Information technology and telecommunications professionals n.e.c.	other workers-office based
2141	Conservation professionals	other workers-non-office based
2142	Environment professionals	other workers-non-office based
2150	Air traffic controllers	other workers-office based
2211	Medical practitioners	healthcare-patient contact
2212	Psychologists	healthcare-patient contact
2213	Pharmacists	healthcare-patient contact
2214	Ophthalmic opticians	healthcare-patient contact
2215	Dental practitioners	healthcare-patient contact
2216	Veterinarians	other workers-non-office based
2217	Medical radiographers	healthcare-patient contact

2218	Podiatrists	healthcare-patient contact
2219	Health professionals n.e.c.	healthcare-patient contact
2221	Physiotherapists	healthcare-patient contact
2222	Occupational therapists	healthcare-patient contact
2223	Speech and language therapists	healthcare-patient contact
2229	Therapy professionals n.e.c.	healthcare-patient contact
2231	Nurses	healthcare-patient contact
2232	Midwives	healthcare-patient contact
2311	Higher education teaching professionals	education
2312	Further education teaching professionals	education
2314	Secondary education teaching professionals	education
2315	Primary and nursery education teaching professionals	education
2316	Special needs education teaching professionals	education
2317	Senior professionals of educational establishments	education
2318	Education advisers and school inspectors	education
2319	Teaching and other educational professionals n.e.c.	education
2412	Barristers and judges	other workers-office based
2413	Solicitors	other workers-office based
2419	Legal professionals n.e.c.	other workers-office based
2421	Chartered and certified accountants	other workers-office based
2423	Management consultants and business analysts	other workers-office based
2424	Business and financial project management professionals	other workers-office based
2425	Actuaries, economists and statisticians	other workers-office based
2426	Business and related research professionals	other workers-office based
2429	Business, research and administrative professionals n.e.c.	other workers-office based
2431	Architects	other workers-office based
2432	Town planning officers	other workers-office based
2433	Quantity surveyors	other workers-office based
2434	Chartered surveyors	other workers-office based
2435	Chartered architectural technologists	other workers-office based
2436	Construction project managers and related professionals	other workers-non-office based
2442	Social workers	social care
2443	Probation officers	social care
2444	Clergy	social care
2449	Welfare professionals n.e.c.	social care
2451	Librarians	other workers-office based
2452	Archivists and curators	other workers-office based
2461	Quality control and planning engineers	other workers-non-office based
2462	Quality assurance and regulatory professionals	other workers-office based
2463	Environmental health professionals	other workers-non-office based
2471	Journalists, newspaper and periodical editors	other workers-office based
2472	Public relations professionals	other workers-office based
2473	Advertising accounts managers and creative directors	other workers-office based

3111	Laboratory technicians	other workers-non-office based
3112	Electrical and electronics technicians	other workers-non-office based
3113	Engineering technicians	other workers-non-office based
3114	Building and civil engineering technicians	other workers-non-office based
3115	Quality assurance technicians	other workers-non-office based
3116	Planning, process and production technicians	other workers-non-office based
3119	Science, engineering and production technicians n.e.c.	other workers-non-office based
3121	Architectural and town planning technicians	other workers-non-office based
3122	Draughtspersons	other workers-non-office based
3131	IT operations technicians	other workers-office based
3132	IT user support technicians	other workers-office based
3213	Paramedics	healthcare-patient contact
3216	Dispensing opticians	healthcare-patient contact
3217	Pharmaceutical technicians	healthcare-patient contact
3218	Medical and dental technicians	healthcare-patient contact
3219	Health associate professionals n.e.c.	healthcare-patient contact
3231	Youth and community workers	social care
3233	Child and early years officers	social care
3234	Housing officers	social care
3235	Counsellors	social care
3239	Welfare and housing associate professionals n.e.c.	social care
3311	NCOs and other ranks	police and protective services
3312	Police officers (sergeant and below)	police and protective services
3313	Fire service officers (watch manager and below)	police and protective services
3314	Prison service officers (below principal officer)	police and protective services
3315	Police community support officers	police and protective services
3319	Protective service associate professionals n.e.c.	police and protective services
3411	Artists	other workers-office based
3412	Authors, writers and translators	other workers-office based
3413	Actors, entertainers and presenters	other workers-non-office based
3414	Dancers and choreographers	other workers-non-office based
3415	Musicians	other workers-non-office based

3416	Arts officers, producers and directors	other workers-non-office based
3417	Photographers, audio-visual and broadcasting equipment operators	other workers-non-office based
3421	Graphic designers	other workers-office based
3422	Product, clothing and related designers	other workers-office based
3441	Sports players	other workers-non-office based
3442	Sports coaches, instructors and officials	other workers-non-office based
3443	Fitness instructors	other workers-non-office based
3511	Air traffic controllers	other workers-office based
3512	Aircraft pilots and flight engineers	transport-public facing
3513	Ship and hovercraft officers	transport-nonpublic facing
3520	Legal associate professionals	other workers-office based
3531	Estimators, valuers and assessors	other workers-office based
3532	Brokers	other workers-office based
3533	Insurance underwriters	other workers-office based
3534	Finance and investment analysts and advisers	other workers-office based
3535	Taxation experts	other workers-office based
3536	Importers and exporters	other workers-office based
3537	Financial and accounting technicians	other workers-office based
3538	Financial accounts managers	other workers-office based
3539	Business and related associate professionals n.e.c.	other workers-office based
3541	Buyers and procurement officers	other workers-office based
3542	Business sales executives	other workers-office based
3543	Marketing associate professionals	other workers-office based
3544	Estate agents and auctioneers	other workers-office based
3545	Sales accounts and business development managers	other workers-office based
3546	Conference and exhibition managers and organisers	other workers-office based
3550	Conservation and environmental associate professionals	other workers-non-office based
3561	Public services associate professionals	other workers-office based
3562	Human resources and industrial relations officers	other workers-office based
3563	Vocational and industrial trainers and instructors	other workers-office based
3564	Careers advisers and vocational guidance specialists	other workers-office based
3565	Inspectors of standards and regulations	other workers-non-office based
3567	Health and safety officers	other workers-non-office based
4112	National government administrative occupations	other workers-office based
4113	Local government administrative occupations	other workers-office based
4114	Officers of non-governmental organisations	other workers-office based
4121	Credit controllers	other workers-office based
4122	Book-keepers, payroll managers and wages clerks	other workers-office based
4123	Bank and post office clerks	other workers-office based
4124	Finance officers	other workers-office based

4129	Financial administrative occupations n.e.c.	other workers-office based
4131	Records clerks and assistants	other workers-office based
4132	Pensions and insurance clerks and assistants	other workers-office based
4133	Stock control clerks and assistants	retail
4134	Transport and distribution clerks and assistants	transport-nonpublic facing
4135	Library clerks and assistants	other workers-office based
4138	Human resources administrative occupations	other workers-office based
4151	Sales administrators	retail
4159	Other administrative occupations n.e.c.	other workers-office based
4161	Office managers	other workers-office based
4162	Office supervisors	other workers-office based
4211	Medical secretaries	healthcare-office based
4212	Legal secretaries	other workers-office based
4213	School secretaries	education
4214	Company secretaries	other workers-office based
4215	Personal assistants and other secretaries	other workers-office based
4216	Receptionists	other workers-office based
4217	Typists and related keyboard occupations	other workers-office based
5111	Farmers	manual
5112	Horticultural trades	manual
5113	Gardeners and landscape gardeners	manual
5114	Groundsmen and greenkeepers	manual
5119	Agricultural and fishing trades n.e.c.	manual
5211	Smiths and forge workers	manual
5212	Moulders, core makers and die casters	manual
5213	Sheet metal workers	manual
5214	Metal plate workers, and riveters	manual
5215	Welding trades	manual
5216	Pipe fitters	manual
5221	Metal machining setters and setter-operators	manual
5222	Tool makers, tool fitters and markers-out	manual
5223	Metal working production and maintenance fitters	manual
5224	Precision instrument makers and repairers	manual
5225	Air-conditioning and refrigeration engineers	manual
5231	Vehicle technicians, mechanics and electricians	manual
5232	Vehicle body builders and repairers	manual
5234	Vehicle paint technicians	manual
5235	Aircraft maintenance and related trades	manual
5236	Boat and ship builders and repairers	manual
5237	Rail and rolling stock builders and repairers	manual
5241	Electricians and electrical fitters	other workers-non-office based
5242	Telecommunications engineers	other workers-non-office based
5244	TV, video and audio engineers	other workers-non-office based

5245	IT engineers	other workers-non-office based
5249	Electrical and electronic trades n.e.c.	other workers-non-office based
5250	Skilled metal, electrical and electronic trades supervisors	manual
5311	Steel erectors	manual
5312	Bricklayers and masons	manual
5313	Roofers, roof tilers and slaters	manual
5314	Plumbers and heating and ventilating engineers	manual
5315	Carpenters and joiners	manual
5316	Glaziers, window fabricators and fitters	manual
5319	Construction and building trades n.e.c.	manual
5321	Plasterers	manual
5322	Floorers and wall tilers	manual
5323	Painters and decorators	manual
5330	Construction and building trades supervisors	manual
5411	Weavers and knitters	manual
5412	Upholsterers	manual
5413	Footwear and leather working trades	manual
5414	Tailors and dressmakers	manual
5419	Textiles, garments and related trades n.e.c.	manual
5421	Pre-press technicians	manual
5422	Printers	manual
5423	Print finishing and binding workers	manual
5431	Butchers	food processing
5432	Bakers and flour confectioners	food processing
5433	Fishmongers and poultry dressers	food processing
5434	Chefs	hospitality
5435	Cooks	hospitality
5436	Catering and bar managers	hospitality
5441	Glass and ceramics makers, decorators and finishers	manual
5442	Furniture makers and other craft woodworkers	manual
5443	Florists	retail
5449	Other skilled trades n.e.c.	manual
6121	Nursery nurses and assistants	education
6122	Childminders and related occupations	education
6123	Playworkers	education
6125	Teaching assistants	education
6126	Educational support assistants	education
6131	Veterinary nurses	other workers-non-office based
6132	Pest control officers	sanitation services
6139	Animal care services occupations n.e.c.	other workers-non-office based
6141	Nursing auxiliaries and assistants	healthcare-patient contact
6142	Ambulance staff (excluding paramedics)	healthcare-patient contact
6143	Dental nurses	healthcare-patient contact

6144	Houseparents and residential wardens	social care
6145	Care workers and home carers	social care
6146	Senior care workers	social care
6147	Care escorts	social care
6148	Undertakers, mortuary and crematorium assistants	social care
6211	Sports and leisure assistants	other workers-non-office based
6212	Travel agents	other workers-office based
6214	Air travel assistants	transport-public facing
6215	Rail travel assistants	transport-public facing
6219	Leisure and travel service occupations n.e.c.	transport-public facing
6221	Hairdressers and barbers	personal care
6222	Beauticians and related occupations	personal care
6231	Housekeepers and related occupations	hospitality
6232	Caretakers	hospitality
6240	Cleaning and housekeeping managers and supervisors	hospitality
7111	Sales and retail assistants	retail
7112	Retail cashiers and check-out operators	retail
7113	Telephone salespersons	other workers-office based
7114	Pharmacy and other dispensing assistants	healthcare-patient contact
7115	Vehicle and parts salespersons and advisers	retail
7121	Collector salespersons and credit agents	other workers-office based
7122	Debt, rent and other cash collectors	other workers-office based
7123	Roundspersons and van salespersons	other workers-non-office based
7124	Market and street traders and assistants	retail
7125	Merchandisers and window dressers	other workers-non-office based
7129	Sales related occupations n.e.c.	retail
7130	Sales supervisors	retail
7211	Call and contact centre occupations	other workers-office based
7213	Telephonists	other workers-office based
7214	Communication operators	other workers-office based
7215	Market research interviewers	other workers-office based
7219	Customer service occupations n.e.c.	other workers-office based
7220	Customer service managers and supervisors	other workers-office based
8111	Food, drink and tobacco process operatives	food processing
8112	Glass and ceramics process operatives	manual
8113	Textile process operatives	manual
8114	Chemical and related process operatives	manual
8115	Rubber process operatives	manual
8116	Plastics process operatives	manual
8117	Metal making and treating process operatives	manual
8118	Electroplaters	manual
8119	Process operatives n.e.c.	manual
8121	Paper and wood machine operatives	manual
8122	Coal mine operatives	manual

8123	Quarry workers and related operatives	manual
8124	Energy plant operatives	manual
8125	Metal working machine operatives	manual
8126	Water and sewerage plant operatives	manual
8127	Printing machine assistants	manual
8129	Plant and machine operatives n.e.c.	manual
8131	Assemblers (electrical and electronic products)	manual
8132	Assemblers (vehicles and metal goods)	manual
8133	Routine inspectors and testers	manual
8134	Weighers, graders and sorters	manual
8135	Tyre, exhaust and windscreen fitters	manual
8137	Sewing machinists	manual
8139	Assemblers and routine operatives n.e.c.	manual
8141	Scaffolders, stagers and riggers	manual
8142	Road construction operatives	manual
8143	Rail construction and maintenance operatives	manual
8149	Construction operatives n.e.c.	manual
8211	Large goods vehicle drivers	transport-nonpublic facing
8212	Van drivers	transport-nonpublic facing
8213	Bus and coach drivers	transport-public facing
8214	Taxi and cab drivers and chauffeurs	transport-public facing
8215	Driving instructors	transport-public facing
8221	Crane drivers	other workers-non-office based
8222	Fork-lift truck drivers	other workers-non-office based
8223	Agricultural machinery drivers	other workers-non-office based
8229	Mobile machine drivers and operatives n.e.c.	other workers-non-office based
8231	Train and tram drivers	transport-nonpublic facing
8232	Marine and waterways transport operatives	transport-nonpublic facing
8233	Air transport operatives	transport-nonpublic facing
8234	Rail transport operatives	transport-nonpublic facing
8239	Other drivers and transport operatives n.e.c.	transport-nonpublic facing
9111	Farm workers	manual
9112	Forestry workers	manual
9119	Fishing and other elementary agriculture occupations n.e.c.	manual
9120	Elementary construction occupations	manual
9132	Industrial cleaning process occupations	sanitation services
9134	Packers, bottlers, canners and fillers	manual
9139	Elementary process plant occupations n.e.c.	manual
9211	Postal workers, mail sorters, messengers and couriers	transport-nonpublic facing
9219	Elementary administration occupations n.e.c.	other workers-office based
9231	Window cleaners	sanitation services
9232	Street cleaners	sanitation services

9233	Cleaners and domestics	sanitation services
9234	Launderers, dry cleaners and pressers	sanitation services
9235	Refuse and salvage occupations	sanitation services
9236	Vehicle valeters and cleaners	sanitation services
9239	Elementary cleaning occupations n.e.c.	sanitation services
9241	Security guards and related occupations	police and protective services
9242	Parking and civil enforcement occupations	police and protective services
9244	School midday and crossing patrol occupations	other workers-non-office based
9249	Elementary security occupations n.e.c.	police and protective services
9251	Shelf fillers	retail
9259	Elementary sales occupations n.e.c.	retail
9260	Elementary storage occupations	manual
9271	Hospital porters	healthcare-patient contact
9272	Kitchen and catering assistants	food processing
9273	Waiters and waitresses	hospitality
9274	Bar staff	hospitality
9275	Leisure and theme park attendants	hospitality
9279	Other elementary services occupations n.e.c.	hospitality

Supplementary Table 1: Occupational groupings used in the analysis.

Occupation	Moderna	Oxford/AstraZenica	Pfizer/BioNTech	Other	Missing
Education	329	7,827	3,709	*	*
%	3	66	31	*	*
Food processing	40	618	383	*	*
%	3.84	59.25	36.72	*	*
Healthcare-office based	*	251	511	*	*
%	*	33	66	*	*
Healthcare-patient contact	54	2,787	7,950	28	27
%	0.5	26	73	0.26	0.25
Hospitality	91	1,354	932	*	*
%	4	57	39	*	*
Manual	329	6,311	3,379	*	*
%	3	63	34	*	*
Other workers-non-office-based	449	6,158	4,417	31	10
%	4	56	40	0.28	0.09
Other workers-office-based	3,028	39,654	25,953	170	63
%	4.4	57.58	37.69	0.25	0.09
Personal care	22	408	267	*	*
%	3.14	58	38	*	*
Police and protective	81	1,975	952	*	*
%	2.69	66	32	*	*
Retail	182	3,346	2,154	*	*
%	3.19	59	38	*	*
Sanitation services	29	1,030	493	*	*
%	1.86	66	32	*	*
Social care	70	2,949	3,362	*	*
%	1.1	46	53	*	*
Transport-nonpublic facing	77	1,946	846	*	*
%	2.68	68	29	*	*
Transport-public facing	22	728	313	*	*
%	2	68	29	*	*
Not working/ student	1,417	39,087	22,170	140	77
%	2	62	35	0.22	0.12
Missing	*	27,963	19,026	118	*
%	*	57	39	0.24	*

Supplementary Table 2: Vaccine type for those receiving a first dose, by occupational group. Due to disclosure rules, counts below 10 are redacted. Values are also redacted where revealing the value would indirectly disclose a count below 10 by subtraction.

Occupation	Moderna	Oxford/AstraZenica	Pfizer/BioNTech	Other	Missing
Education	325	7,798	3,679	11	11
%	3	66	31	0.09	0.09
Food processing	39	614	374	*	*
%	3.79	59.73	36.38	*	*
Healthcare-office based	*	249	509	*	*
%	*	33	66	*	*
Healthcare-patient contact	53	2,756	7,905	22	35
%	0.49	26	73	0.2	0.32
Hospitality	85	1,350	913	*	*
%	4	57	39	*	*
Manual	324	6,283	3,292	*	*
%	3	63	33	*	*
Other workers-non-office-based	441	6,135	4,356	24	14
%	4	56	40	0.22	0.13
Other workers-office-based	2,984	39,543	25,648	110	124
%	4.36	57.8	37.49	0.16	0.18
Personal care	22	404	262	*	*
%	3.18	58	38	*	*
Police and protective	76	1,965	943	*	*
%	2.54	66	32	*	*
Retail	180	3,327	2,118	*	*
%	3.19	59	38	*	*
Sanitation services	30	1,023	482	*	*
%	1.95	66	31	*	*
Social care	69	2,931	3,334	*	*
%	1.09	46	53	*	*
Transport-nonpublic facing	75	1,938	832	*	*
%	2.63	68	29	*	*
Transport-public facing	19	726	308	*	*
%	2	69	29	*	*
Not working/ student	1,372	38,738	21,671	97	139
%	2	62	35	0.16	0.22
Missing	*	27,848	18,733	81	*
%	*	57	39	0.17	*

Supplementary Table 3: Vaccine type for those receiving a second dose, by occupational group. Due to disclosure rules, values are redacted when counts are below 10. Values are also redacted where revealing the value would indirectly disclose a count below 10 by subtraction.

**Occupation**	Moderna	Oxford/AstraZenica	Pfizer/BioNTech	Other
Education	3,460	*	7,528	*
%	31	*	68	*
Food processing	260	*	600	*
%	30.23	*	69.77	*
Healthcare-office based	82	*	658	*
%	11.08	*	89	*
Healthcare-patient contact	829	*	9,225	*
%	8.23	*	92	*
Hospitality	597	*	1,418	*
%	30	*	70	*
Manual	2,833	*	5,812	*
%	33	*	67	*
Other workers-non-office-based	3,283	*	6,672	*
%	33	*	67	*
Other workers-office-based	20,677	51	42,692	15
%	32.6	0.08	67.3	0.02
Personal care	176	*	409	*
%	30.03	*	70	*
Police and protective	912	*	1,776	*
%	33.9	*	66	*
Retail	1,575	*	3,380	*
%	31.74	*	68	*
Sanitation services	380	*	987	*
%	27.78	*	72	*
Social care	1,025	*	4,767	*
%	17.68	*	82	*
Transport-nonpublic facing	846	*	1,697	*
%	33.27	*	67	*
Transport-public facing	274	*	672	*
%	29	*	71	*
Not working/ student	15,073	104	41,211	18
%	27	0	73	0.03
Missing	13,708	45	30,710	15
%	31	0	69	0.03

Supplementary Table 4: Vaccine type for those receiving a third dose, by occupational group. Due to disclosure rules, values are redacted when counts are below 10. Values are also redacted where revealing the value would indirectly disclose a count below 10 by subtraction.

Number of vaccines received	Hazard Ratio (95% CI)
0	Ref
1	0.80 (0.76 to 0.85)
2	0.71 (0.68 to 0.75)
3	0.40 (0.38 to 0.41)

Supplementary Table 5: Hazard ratios (95% CIs) for number of vaccines received in relation to infection with SARS-CoV-2. Based on n = 256,598 individuals. Adjusted for occupation, age, sex, ethnicity, deprivation, region, urban or rural area, household size, and presence of pre-existing health conditions.

	Number of infections			
**Occupation**	**0**	**1**	**2**	**3**
Education	8,846	3,209	76	0
	72.92	26.45	0.63	0
Food processing	830	246	*	*
	76.85	22.78	*	*
Healthcare-office based	612	162	*	*
	78.76	20.85	*	*
Healthcare-patient contact	8,544	2,379	47	0
	77.89	21.69	0.43	0
Hospitality	1,919	528	15	0
	77.94	21.45	0.61	0
Manual	8,080	2,265	42	0
	77.79	21.81	0.4	0
Other workers-non-office-based	8,892	2,403	56	0
	78.34	21.17	0.49	0
Other workers-office-based	54,857	15,027	*	*
	78.13	21.4	*	*
Personal care	539	189	*	*
	73.63	25.82	*	*
Police and protective	2,327	733	16	0
	75.65	23.83	0.52	0
Retail	4,598	1,257	20	0
	78.26	21.4	0.34	0
Sanitation services	1,245	357	*	*
	77.33	22.17	*	*
Social care	5,007	1,481	45	0
	76.64	22.67	0.69	0
Transport-nonpublic facing	2,318	655	17	0
	77.53	21.91	0.57	0
Transport-public facing	833	246	*	*
	76.56	22.61	*	*
Not working/student	52,214	12,749	*	*
	80.04	19.54	*	*
Missing/incomplete	38,479	11,335	276	0
	76.82	22.63	0.55	0

Supplementary Table 6: Number of infections by occupational group. Due to disclosure rules, values are redacted when counts are below 10. Values are also redacted where revealing the value would indirectly disclose a count below 10 by subtraction.

	Model 1	Model 2	Model 3
Education	1.272 [1.226,1.320]	1.252 [1.207,1.299]	1.250 [1.204,1.297]
Food processing	1.049 [0.931,1.181]	1.041 [0.924,1.173]	0.984 [0.874,1.109]
Healthcare-office based	0.901 [0.775,1.049]	0.906 [0.778,1.054]	0.950 [0.816,1.105]
Healthcare-patient contact	1.016 [0.974,1.059]	1.016 [0.974,1.060]	1.071 [1.027,1.117]
Hospitality	1.084 [1.006,1.168]	1.081 [1.003,1.165]	1.033 [0.959,1.114]
Manual	0.993 [0.951,1.036]	0.995 [0.953,1.039]	0.947 [0.907,0.990]
Other workers-non-office based	0.991 [0.952,1.032]	0.994 [0.955,1.036]	0.982 [0.943,1.023]
Other workers-office based	1 [1,1]	1 [1,1]	1 [1,1]
Personal care	1.155 [1.011,1.319]	1.150 [1.006,1.313]	1.076 [0.942,1.229]
Police and protective services	1.101 [1.025,1.182]	1.113 [1.036,1.196]	1.098 [1.022,1.179]
Retail	0.991 [0.937,1.048]	0.992 [0.938,1.049]	0.959 [0.906,1.014]
Sanitation services	1.032 [0.934,1.141]	1.031 [0.933,1.140]	0.986 [0.892,1.091]
Social care	1.104 [1.048,1.162]	1.100 [1.045,1.158]	1.111 [1.055,1.169]
Transport-nonpublic facing	1.057 [0.980,1.139]	1.058 [0.981,1.140]	1.007 [0.934,1.086]
Transport-public facing	1.121 [1.001,1.256]	1.116 [0.996,1.250]	1.082 [0.966,1.213]

Not working/student	0.939 [0.916,0.962]	0.939 [0.916,0.963]	0.906 [0.884,0.929]
Missing/incomplete	0.887 [0.866,0.909]	0.886 [0.865,0.908]	0.881 [0.860,0.903]

Supplementary Table 7: Hazard ratios (95% CIs) corresponding to occupational group in relation to infection with SARS-CoV-2. Based on n = 256,598 individuals. Model 1: Adjusted for age and sex. Model 2: Additionally adjusted for ethnicity, deprivation, region, urban or rural area, household size, and presence of pre-existing health conditions. Model 3: Additionally adjusted for number of vaccines received.

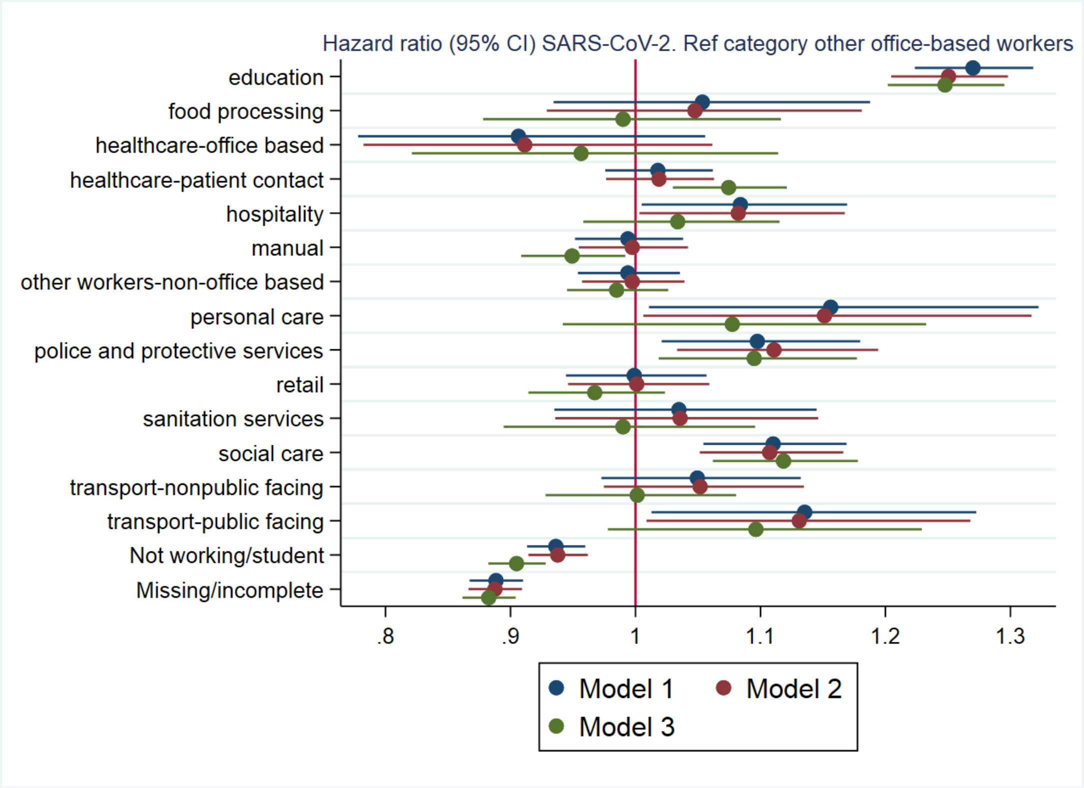

Supplementary Figure 1: Sensitivity analysis, where a 6-month gap and negative PCR test is required between positive tests to register a new infection. Hazard ratios (95% CIs) corresponding to occupational group in relation to infection with SARS-CoV-2. Based on n = 256,598 individuals. Model 1: Adjusted for age and sex. Model 2: Additionally adjusted for ethnicity, deprivation, region, urban or rural area, household size, and presence of pre-existing health conditions. Model 3: Additionally adjusted for number of vaccines received.

	Model 1	Model 2	Model 3
education	1.270<br>[1.224,1.318]	1.250<br>[1.205,1.298]	1.248<br>[1.202,1.295]
food processing	1.054<br>[0.934,1.188]	1.047<br>[0.929,1.181]	0.990<br>[0.878,1.116]
healthcare-office based	0.906<br>[0.778,1.056]	0.911<br>[0.782,1.062]	0.956<br>[0.821,1.114]
healthcare-patient contact	1.018<br>[0.976,1.062]	1.019<br>[0.977,1.063]	1.075<br>[1.030,1.121]
hospitality	1.084<br>[1.005,1.169]	1.082<br>[1.003,1.168]	1.034<br>[0.958,1.115]
manual	0.994<br>[0.952,1.038]	0.997<br>[0.955,1.042]	0.949<br>[0.909,0.992]
other workers-non-office based	0.994<br>[0.954,1.036]	0.997<br>[0.957,1.039]	0.985<br>[0.945,1.026]
other workers-office based	1<br>[1,1]	1<br>[1,1]	1<br>[1,1]
personal care	1.156<br>[1.011,1.323]	1.151<br>[1.006,1.317]	1.077<br>[0.942,1.233]
police and protective services	1.098<br>[1.021,1.180]	1.111<br>[1.033,1.194]	1.095<br>[1.019,1.177]
retail	0.999<br>[0.944,1.057]	1.001<br>[0.946,1.059]	0.967<br>[0.914,1.024]
sanitation services	1.035<br>[0.935,1.145]	1.036<br>[0.936,1.146]	0.990<br>[0.895,1.096]
social care	1.110<br>[1.054,1.169]	1.107<br>[1.051,1.166]	1.118<br>[1.062,1.178]
transport-nonpublic facing	1.049<br>[0.973,1.132]	1.052<br>[0.975,1.135]	1.001<br>[0.928,1.081]
transport-public facing	1.135<br>[1.013,1.273]	1.131<br>[1.009,1.268]	1.096<br>[0.978,1.229]
Not	0.936	0.938	0.905

working/student	[0.913,0.960]	[0.914,0.962]	[0.882,0.928]
Missing/incomplete	0.888 [0.867,0.910]	0.887 [0.866,0.909]	0.883 [0.862,0.904]

Supplementary Table 8: Sensitivity analysis, where a 6-month gap and negative PCR test is required between positive tests to register a new infection. Hazard ratios (95% CIs) corresponding to occupational group in relation to infection with SARS-CoV-2. Based on n = 256,598 individuals. Model 1: Adjusted for age and sex. Model 2: Additionally adjusted for ethnicity, deprivation, region, urban or rural area, household size, and presence of pre-existing health conditions. Model 3: Additionally adjusted for number of vaccines received.
